# Elevated Endogenous Psychedelic Bufotenine in the Urine of Patients Diagnosed With a Mental Illness: A Systematic Review

**DOI:** 10.7759/cureus.84510

**Published:** 2025-05-20

**Authors:** Alistair J Clarke

**Affiliations:** 1 General Internal Medicine, Cambridge University Hospitals NHS Foundation Trust, Cambridge, GBR

**Keywords:** autism spectrum disorder (asd), bufotenine, mental illness, psychedelic, schizophrenia and other psychotic disorders, tryptamines, urine

## Abstract

The objective of this systematic review was to determine if higher concentrations of urinary bufotenine occur in patients with a mental illness compared to healthy controls. Bufotenine is an alkaloid with psychedelic and psychoactive properties that closely resembles the structure of serotonin. Bufotenine naturally occurs in Anadenanthera seeds and is secreted along with 5-methoxy-N, N-dimethyltryptamine (5-MeO-DMT) in the venom of the Bufo alvarius toad. There is evidence that bufotenine is produced endogenously in human subjects and is associated with mental illness, in particular schizophrenia and autism spectrum disorder. A total of eight full-text papers were included in the review, with a total of 609 participants, of whom 275 had a psychiatric diagnosis and 318 were healthy controls. In 226 out of 275 (82%) patients with a mental illness, urinary bufotenine was detected, compared with only 92 of 318 (29%) non-psychiatric subjects. The findings in the studies were somewhat heterogeneous; five out of eight studies found higher concentrations of urinary bufotenine in those with a mental illness than those without, of which three studies showed a statistically significant difference. One study failed to detect any urinary bufotenine in any of their subjects, and two out of the eight found a significant overlap of urinary bufotenine concentrations between those with a mental illness and healthy controls. Consequently, at present, it is likely too early to propose bufotenine as a possible biomarker for mental illnesses. Yet, further research is certainly merited, especially given that endogenously produced bufotenine could be implicated in the pathophysiology of mental illnesses.

## Introduction and background

Grammenos et al. [1] summarize the transmethylation hypothesis, suggesting that environmental stress, coupled with the abnormal metabolism of tryptophan-derived neurotransmitters, can lead to the synthesis of endogenous psychedelic tryptamines. They speculate that such tryptamines are implicated in the pathophysiology of the positive symptoms of psychosis. Indeed, studies have shown that levels of N, N-dimethyltryptamine (DMT) increase in rodent brains under conditions of stress [2]. Moreover, exogenously administered DMT, as well as other classic psychedelic compounds such as psilocybin and lysergic acid diethylamide (LSD), are known to act as 5-HT2a receptor agonists, producing potent changes in phenomenology, replicating the positive symptoms of psychosis [1,3].

Barker et al. [4] conducted a review on the presence of endogenous psychedelic tryptamines in humans, including DMT, bufotenine (5-hydroxy-DMT), and 5-methoxy-DMT (5-MeO-DMT). The review found compelling evidence that these psychedelic tryptamines are produced endogenously, with detectable levels in the blood, cerebrospinal fluid, and urine of both healthy individuals and those with mental illness.

Exogenous bufotenine is known to have psychedelic properties, leading to changes in emotional state and visual hallucinations [5,6]. McLeod and Sitaram [5] consider bufotenine to be psychotomimetic, meaning it mimics psychotic-like symptoms. The exact mechanism by which bufotenine exhibits a hallucinogenic effect is unclear. However, bufotenine shares structural similarities with other psychedelic compounds such as DMT and 5-MeO-DMT and is an isomer of psilocin [7]. Similar to other psychedelics such as LSD, psilocybin, and psilocin, bufotenine's hallucinogenic mode of action may be attributed to its role as a 5-HT2a receptor agonist [7-9].

The existence of endogenous production of bufotenine, DMT, and 5-MeO-DMT certainly supports the transmethylation hypothesis [1,4]. Moreover, DMT is known to be produced via methylation, a process that involves the addition of methyl groups to tryptamine, which is catalyzed by the enzyme indolethylamine-N-methyltransferase (INMT) [1]. Endogenous bufotenine production may follow a similar metabolic pathway.

As yet, no systematic review has compared urinary bufotenine levels in healthy controls and psychiatric patient populations. Consequently, this review aims to investigate the following question: “Are concentrations of urinary bufotenine in patients with a mental illness significantly elevated when compared to healthy controls?” To formulate this research question, the Population, Indicator, Comparison, and Outcome (PICO) framework was utilized as outlined in Table 1. The importance of this review lies in the utility of investigating bufotenine’s potential as a biomarker of psychiatric disease or even its possible role in the pathophysiology of mental illness.

**Table 1:** A PICO description applied in this review. PICO: Population, Indicator, Comparison, and Outcome; EMBASE: Excerpta Medica Database; MEDLINE: Medical Literature Analysis and Retrieval System Online

PICO categories	Description	EMBASE keywords	Mesh terms	MEDLINE search terms
Population	Patients with a mental health condition	Mental Disease	Mental Disorders	Mental health or mental illness or mental disorder, or psychiatric illness
Indicator	Measurement of urinary bufotenine concentration			Urine
Comparison	Healthy volunteers			
Outcome	Urinary bufotenine is elevated in patients with mental health conditions	Bufotenine	Bufotenin	Tryptamines OR indoleamines OR Bufotenin OR Bufotenine OR mappine OR 5-hydroxy-N, N-dimethyletryptamine OR N, N-dimethyl-serotonin or N, N-dimethyl-5-hydroxytryptamine

## Review

Methods

Search Strategy and Selection Criteria 

The search strategy included keyword searches on Excerpta Medica Database (EMBASE), an advanced search on Medical Literature Analysis and Retrieval System Online (MEDLINE), and Medical Subject Headings (MeSH) searches on MEDLINE.

Search 1 was completed on MEDLINE as an advanced search (Tryptamines OR indoleamines OR Bufotenin OR Bufotenine OR mappine OR 5-hydroxy-N,N-dimethyletryptamine OR N,N-dimethyl-serotonin or N,N-dimethyl-5-hydroxytryptamine) AND (mental health OR mental illness OR mental disorder OR psychiatric illness) AND urine, which yielded 20 results.

Search 2 was completed on MEDLINE Medical Subject Headings (MH "Mental Disorders+") AND (MH "Bufotenin+"), yielding 51 from MEDLINE.

Search 3 was completed on EMBASE ('mental disease'/exp OR 'mental disease') AND ('bufotenine'/exp OR bufotenine) AND ('urine'/exp OR urine) and gave 28 results.

**Figure 1:**
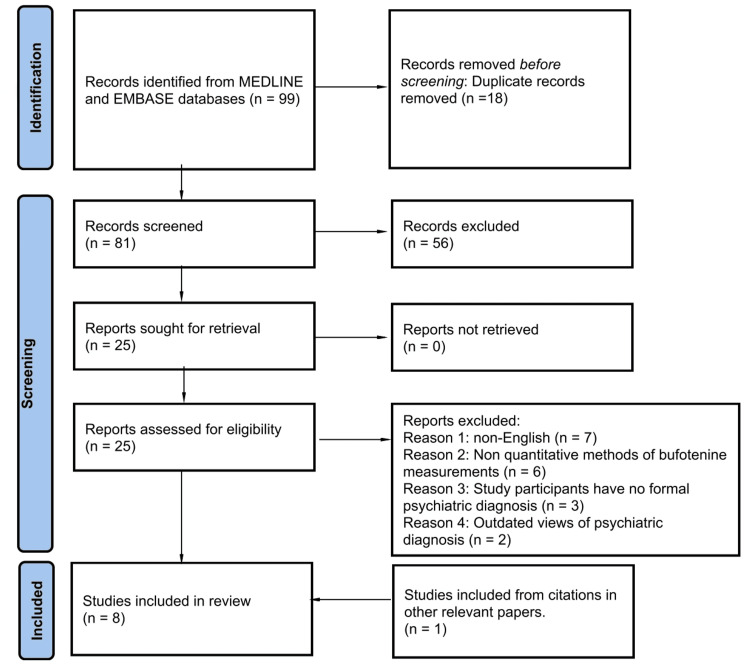
Flow diagram of selection criteria. Adapted from the PRISMA 2020 statement from Page et al. [12]. EMBASE: Excerpta Medica Database; MEDLINE: Medical Literature Analysis and Retrieval System Online; PRISMA: Preferred Reporting Items for Systematic reviews and Meta-Analyses

Combining the three search strategies yielded a total of 99 papers, 18 of which were duplicates, leaving 81 unique papers for screening. Of these 81 papers, 25 were assessed against the inclusion criteria, summarized in Figure 1. Seven of these papers were found to be appropriate for addressing the research question. One of these seven papers, Emanuele et al. [10], cited Forsström et al. [11]; the latter paper was not captured in the initial search but was deemed to fit the inclusion criteria. Hence, this brought the total number of studies incorporated in the review to eight. Ultimately yielding a total of 275 subjects with a psychiatric diagnosis and 318 controls across the eight studies included. 

Studies conducted on animals and papers not originally authored in English were pragmatically excluded due to the unavailability of reliable translation methods. There were no restrictions based on age, ethnicity, country, or sex. In line with the research question, only papers reporting quantitative measurements of urinary bufotenine were included. This approach allows for comparisons with control groups and across studies. As a result, several older papers that ostensibly appeared relevant were excluded. Fischer et al. [13], Narasimhachari et al. [14], and Brune et al. [15] claimed to have detected bufotenine in the urine of individuals with schizophrenia but employed non-quantitative chromatography methods and provided no concentration level.

In addition, whilst there was no time criterion, the study participants must have a psychiatric diagnosis that aligns with the modern-day understanding of mental illnesses. Therefore, studies that include outdated conceptions of psychiatric disorders not consistent with the Diagnostic and Statistical Manual of Mental Disorders, Fifth Edition (DSM-5), or the International Classification of Diseases, 11th Revision (ICD-11), will be excluded [16,17]. Consequently, Kärkkäinen et al. [18], Räisänen et al. [19], and Bastos et al. [20] detected bufotenine in their study subjects, but the participants had no confirmed mental illness, and these papers were thus excluded. Additionally, Saavedra et al. [21] and Sireix et al. [22], despite employing quantitative methods, included individuals with redundant diagnoses of hysteria and homosexuality.

Results

In their 2010 cross-sectional study, Emanuele et al. [10] investigated the levels of urinary bufotenine in individuals with severe autism spectrum disorder (ASD) (n=15, 10 males and five females), schizophrenia (n=15, 10 males and five females), and healthy controls (n=18). The study aimed to compare the levels of urinary bufotenine in patients with a mental illness and healthy controls. They reported urinary bufotenine levels were significantly higher in patients with a mental illness compared to healthy controls. Specifically, the mean bufotenine level was found to be 3.30 ± 0.49 μg/L (p<0.05) in individuals with ASD and 4.39 ± 0.43 μg/L (p<0.001) in those with schizophrenia, whereas the control group had a mean urinary bufotenine level of 1.53 ± 0.30 μg/L.

In a cross-sectional study by Forsström et al. [11], high-performance liquid chromatography was used to analyze bufotenine in the urine of surgical, medical, and psychiatric patients. However, urinary collections were not controlled for the time of day. Urinary bufotenine was detected in 48% (14/29) of psychiatric patients, 30% (7/23) of surgical patients, and 15% (2/13) of medical patients. The authors found a statistically significant difference in bufotenine detection between medical patients (2/13) and psychiatric patients (14/29) (p<0.001), with greater detection in this last group. It was also noted that one surgical patient had higher levels of bufotenine than any of the psychiatric patients.

Takeda et al. [23] conducted a cross-sectional study and discovered urinary bufotenine in 50 out of 65 patients with ASD, 15 out of 16 patients with depression, and 13 out of 15 patients with schizophrenia. In comparison, only two out of 200 healthy controls tested positive for urinary bufotenine. The mean bufotenine level for the 13 schizophrenic patients in whom bufotenine was detected was 289.9 ± 487.6 ng/mg (nanograms of bufotenine per mg of urinary creatinine), which was an order of magnitude higher than any other participant group. The study also measured urinary serotonin levels, which were found to be markedly higher in schizophrenic participants. Despite these results, no statistical significance was calculated for any data in this study.

Kärkkäinen et al. [24] conducted a two-week longitudinal study comparing urinary excretion of bufotenine in psychiatric inpatients and healthy controls. Morning urine samples were obtained from 75 patients on admission to the hospital and two weeks later. The study found the median urinary bufotenine levels remained stable at 9.2 nmol/g (nmol bufotenine per g of creatinine) and 9.6 nmol/g at admission and two-week follow-up, respectively. By comparison, 51 healthy controls had a median bufotenine level of 1.8 nmol/g. They reported significantly higher median urinary bufotenine levels (p=0.001) in psychiatric patients compared to controls. Although the study design was not controlled for medication or specific psychiatric diagnosis, Kärkkäinen et al. [24] did examine the link between medication and urinary bufotenine levels. The study found significantly higher bufotenine levels in patients taking monoamine oxidase inhibitors (MAOIs) or selective serotonin reuptake inhibitors (SSRIs) (p=0.0035). Only three patients were drug-free during the entire study, which is a potential confounding factor in interpreting the results.

Cottrell et al. [25] performed a small cross-sectional study utilizing gas-liquid chromatography (GLC) to analyze a bufotenine-like substance level from 24-hour urine collections in psychiatric inpatients (n=20). Mean bufotenine-like substance levels were found to be 29 nmol/24 hrs, compared to 0 nmol/24 hr in healthy controls (n=2). While the primary focus of the research was to analyze the urine of individuals with schizophrenia, the study also included participants with bipolar disorder and personality disorders. Of the nine patients whose bufotenine-like levels were greater than 10 nmol/24 hr, all had a diagnosis of schizophrenia. However, it is important to note that the study analyzed a bufotenine-like substance, indicating the researchers' uncertainty in the sensitivity of their analysis techniques.

Huszka et al. [26] conducted a study on seven female patients with schizophrenia. The study consisted of several phases, during which all patients, except for an initial three-week baseline period, were administered an MAO inhibitor alongside receiving tryptophan, glycine, or sucrose placebo. Surprisingly, no psychedelic tryptamines were detected in any of the patients' urine during any phase of the study. This finding contradicts the Kärkkäinen et al. [24] study, which found the highest levels of urinary bufotenine in a patient taking an MAO inhibitor. Furthermore, Huszka et al. [26] deprived patients of their antipsychotic medications, which, although desirable as a control variable, raises ethical concerns.

Carpenter et al. [27] conducted a study to measure urinary bufotenine, DMT, and INMT enzyme activity in acute admission schizophrenic patients. The study involved a patient group (n=26) and a control group (n=7), and 24-hour urine collections were taken from all subjects and analyzed using two methods: thin-layer chromatography on silica gel and gas chromatography-mass spectrometry (GC-MS). Regarding bufotenine, thin-layer chromatography revealed mean urinary levels of 1.67 µg/24 hr and 1.73 µg/24 hr in the patient and control groups, respectively. Similarly, using GC-MS, they found 1.14 µg/24 hr in patients and 1.71 µg/24 hr in controls. An unpaired t-test showed no significant difference between the groups (p<0.25). Additionally, bufotenine was detected significantly more frequently in controls than in patients (p<0.05).

Fischer et al. [28] measured urinary bufotenine concentrations in healthy controls (n=4), chronic schizophrenic patients (n=4), and acute drug-free schizophrenic patients (n=4). To obtain the free form of bufotenine, they utilized glucuronidase to hydrolyze the conjugated form. By comparing the spectroscopic analysis of treated and untreated samples, both free and total bufotenine concentrations could be determined. The mean values of free bufotenine were not significantly different between the chronic schizophrenic group and healthy controls, but the acute schizophrenic group showed significant differences in mean free bufotenine levels (p between 0.01-0.001). On the other hand, mean total bufotenine levels differed significantly between controls (9.3 µg%) and chronic schizophrenics (17.3 µg%) (p=0.01). The acute group had the highest mean total bufotenine levels at 28.9 µg%. It appears that chronic schizophrenic patients tend to excrete a greater proportion of bufotenine in its conjugate form compared to the acute group, but a larger sample size is necessary to draw any definitive conclusions. Table 2 summarizes the aforementioned results and analysis methods for each study.

**Table 2:** Summary of results. 5-MeO-DMT: 5-methoxy-N, N-dimethyltryptamine; ASD: autism spectrum disorder

Authors and year	Sample size	Study design	Collection method	Analysis method	Results
Emanuele et al. [10] 2010	Patients (n=30) and controls (n=18)	Cross-sectional	Mid-stream urine collection, frozen at -40ºC for analysis	High-performance liquid chromatography-mass spectrometry (HPLC-MS) assay	Urinary bufotenine levels were markedly increased in autistic spectrum disorder subjects (3.30 ± 0.49 μg/L, p<0.05) and patients with schizophrenia (4.39 ± 0.43 μg/L, p<0.001) compared with controls (1.53 ± 0.30 μg/L)
Forsström et al. [11] 2001	Patients (n=29) and controls (n=36)	Cross-sectional	Urine from patients, either AM or PM sample, frozen to -20 ºC	High-performance liquid chromatography-electrospray ionisation-mass spectrometry (HPLC/ESI-MS)	Urinary bufotenine was detected in 48% of psychiatric (14/29), 30% of surgical (7/23), and 15% of medical patients (2/13). Concentration ranges from 0.81-24.9 µg/L, 0.43–33.57 µg/L, and 0.48-7.7 µg/L in the psychiatric, surgical, and medical groups, respectively.
Takeda et al. [23] 1995	Patients (n=96) and controls (n=200)	Cross-sectional	First AM voided urine, where possible	High-performance liquid chromatography (HPLC) with electrochemical detection	Urinary bufotenine was found in 50/65 patients with ASD, 15/16 patients with depression, and 13/15 patients with schizophrenia, compared with only 2/200 healthy controls. The mean bufotenine level in schizophrenic subjects was 289.9 ± 487.6 ng/mg (nanograms of bufotenine per mg of urinary creatinine), a power of 10 higher than any other patient group.
Kärkkäinen et al. [24] 1988	Patients (n=75) and controls (n=51)	Longitudinal 2-week study	Morning urine collection on admission and 2 weeks hence	Gas chromatography-mass spectrometry (GC-MS)	n=75 psychiatric inpatients, median bufotenine levels 9.2 nmol/g on admission, 9.6 nmol/g at 2 weeks, n=51 control 1.8 nmol/g.
Cottrell et al. [25] 1977	Patients (n=20) and controls (n=2)	Cross-sectional	24-hour urine collection	Gas-liquid chromatography	n=20 psychiatric inpatients, mean bufotenine-like substance levels 29 nmol/24 hrs, compared to 0 nmol/24 hr in n=2 healthy controls
Huszka et al. [26] 1976	Patients (n=7) and controls (n=0)	Multi-phase longitudinal	24-hour urinary collection	Gas-liquid chromatography	n=7 schizophrenic women, no urinary tryptamines detected (DMT, 5-MeO-DMT, bufotenine)
Carpenter et al. [27] 1975	Patients (n=26) and controls (n=7)	Cross-sectional	24-hour urine collection	Thin-layer silica gel chromatography and gas chromatography-mass spectrometry (GC-MS)	n=26 acute schizophrenic patients, n=7 controls. Thin layer chromatography, mean urinary bufotenine 1.67 µg/24hr and 1.73 µg/24hr in the patient and control groups, respectively. GC-MS yielded 1.14 µg/24hr in patients and 1.71µg/24hr urinary bufotenine in controls (difference not significant, p<0.25). Bufotenine was detected significantly more often in controls than in patients, p<0.05
Fischer et al. [28] 1971	Patients (n=8) and controls (n=4)	Cross-sectional	First-morning urine collection	Spectrophotometry	n=4 acute untreated schizophrenics, n=4 chronic schizophrenics, n=4 controls - mean total urinary bufotenine 28.9 µg%, 17.3 µg% and 9.3 µg%. The difference between the chronic and control group was significant, p=0.01

Discussion

Only Fischer et al. [28] specifically addressed the excretion of bufotenine through the conjugation of free amine. According to their estimation, one-third of bufotenine is excreted as free amine, and without the hydrolysis of the bufotenine-glucuronic acid conjugate, the concentration of bufotenine may be underestimated. Whilst an in-depth evaluation of analytical chemistry techniques is beyond the scope of this review, across the eight studies, there was limited consistency in the methods used to determine the presence and concentration of bufotenine, making direct comparison of results difficult.

After aggregating data from all eight studies, it was observed that 226 out of 275 (82%) patients with a diagnosis of mental illness tested positive for urinary bufotenine, whereas only 92 of 318 (29%) non-psychiatric subjects tested positive. However, this data is significantly skewed by Takeda et al. [23], who found only 2/200 controls positive for urinary bufotenine. When this data is excluded, 76% of controls tested positive for urinary bufotenine. This is surprising because bufotenine is commonly found at lower concentrations in healthy controls [10,11,24,27], and Takeda et al. [23] claim to have used highly sensitive methods with an ability to detect bufotenine levels as low as 50 ng/ml.

The concentration of urinary bufotenine is more salient than its detection rate. However, comparing concentrations across studies is challenging due to variations in analytical chemistry techniques, collection methods, and reporting units. Nevertheless, trends can be aggregated. Emanuele et al. [10] and Fischer et al. [28] found statistically significant (p≤0.05) higher mean urinary bufotenine concentrations in subjects with mental illness compared to those without. Takeda et al. [23] and Cottrell et al. [25] also support this finding, but did not provide data pertaining to statistical significance. Similarly, Kärkkäinen et al. [24] reported significantly higher median urinary bufotenine levels (p=0.001) in psychiatric patients compared to controls. Forsström et al. [11] only provided the ranges of bufotenine levels detected and did not report mean or median levels. Carpenter et al. [27] found no significant difference (p<0.25) in mean urinary bufotenine between controls and psychiatric patients. Finally, Huszka et al. [26] did not detect urinary bufotenine in their seven schizophrenic subjects.

All the papers included in this review suffer from small sample sizes, potentially leading to underpowered studies. In addition, a major potential confounding factor is the concurrent use of psychotropic medications in the patient groups. For example, Kärkkäinen et al. [24] found significantly higher urinary bufotenine levels in patients taking MAOIs or SSRIs (p=0.0035). Conversely, Huszka et al. [26] found no urinary bufotenine in patients explicitly administered MAOIs, although this may have been offset by the fact that these patients were also placed on a low serotonin diet. Only Carpenter et al. [27] and Emanuele et al. [10] ensured all patients were either medication-naive or had ceased medication before the study. Furthermore, both these studies carefully screened controls to exclude individuals with a personal or family history of mental illness. Unfortunately, Forsström et al. [11] did not delineate patients by psychiatric diagnosis, and it does not appear that the surgical and medical patient groups were screened for any psychiatric comorbidities.

Regarding study design, only Cottrell et al. [25] and Emanuele et al. [10] explicitly included blinding elements by withholding clinical details from laboratory personnel. The latter study also utilized independent psychiatrists for participant diagnosis. In contrast, Carpenter et al. [27] relied on the clinical study lead for diagnosis, leaving open the possibility of bias. The method of clinical diagnosis was not explicitly stated in the other studies included in the review, raising questions concerning the objectivity of psychiatric diagnoses [11,23,24,26,28]. This also makes direct comparisons of subjects between studies less reliable. Additionally, a further issue in study comparison and design is the differing methods of urine collection. Some studies analyzed single urine void collections [10,11,23,24,28], while others analyzed 24-hour collections [25-27]. As bufotenine excretion could vary over time, single-voided specimens may not account for these fluctuations.

Of all the studies, Emanuele et al. [10] appeared to conduct the highest quality study that included the careful screening of controls, blinding, and independent diagnoses of psychiatric patients, and ensured all participants were drug-free during the study. Their findings showed urinary bufotenine levels were markedly increased in subjects with ASD (p<0.05) and patients with schizophrenia (p<0.001) compared with controls. Considering the robustness of their methodology, it is surprising that research in this domain remains limited. Additional studies are certainly necessary to investigate the mechanisms underlying these findings and their clinical significance. 

In conducting this systematic review, it is important to acknowledge certain limitations, notably the potential for publication bias stemming from the inclusion of studies solely in English. This language bias may have excluded relevant research published in other languages, thereby limiting the scope of the analysis. Additionally, the inability to perform a meta-analysis was driven by significant clinical and methodological heterogeneity among the studies, coupled with small sample sizes. As a strength, it should be noted that this is the first systematic review to date on this topic.

## Conclusions

This review aimed to answer the question, “Are concentrations of urinary bufotenine in patients with mental illness significantly elevated when compared to healthy controls?” The current and admittedly limited available evidence suggests that bufotenine levels are not consistently or significantly elevated in mental illness. This is partly due to poor study design, small sample sizes, lack of standardization in selecting study participants, and variable analysis methods. Additionally, the aggregation of results revealed considerable overlap in bufotenine levels in the urine of psychiatric patients and controls. 

However, it is important to note that certain studies have highlighted an association between elevated urinary bufotenine levels and specific psychiatric conditions, such as psychotic disorders and ASD. This apparent contradiction may stem from differences in study methodologies, participant demographics, or other confounding factors. Nevertheless, bufotenine's known action as a 5HT-2A agonist provides a plausible mechanism for its potential role in contributing to positive psychotic symptoms if endogenously produced. This underscores the necessity for further research to clarify the nature and extent of this association.

Regrettably, biomarkers have limited current utility in the diagnosis of mental illness; clinicians depend almost exclusively on the psychiatric history and mental state exam. This likely reflects our still limited understanding of the pathophysiology of mental illness. Clearly, a better understanding of the biological basis of mental disorders will be critical for the development of new diagnostic tools and new pharmacological targets.
